# Ague as a Providential Dispensation

**Published:** 1875-04-01

**Authors:** 


### AGUE AS A PROVIDENTIAL DISPENSATION.

THE wisdom and kindness of Providence in the allotment of disease, is not always appreciated by the sufferers themselves, at least. Mark Twain in his narrative of "Old Times on the Mississippi," in the March *Atlantic*, makes clear, however, the happy and providential aspect of one disease, at least. Describing graphically the life and aspect on the low bottom lands bordering the river, during a big rise and overflow, he says:

"There were crazy rail-fences sticking a foot or two above the water, with one or two jeans-clad, chillsrocked, yellow-faced, male miserables roasting on the top-rail, elbows on knees, jaws in hands, grinding tobacco, and discharging the result at floating chips, through crevices left by last milk-teeth; while the rest of the family, and the few farm animals, were huddled together in an empty wood-flat, riding at her moorings close at hand. In this flat-boat, the family would have to cook, and eat, and sleep, for a lesser or greater number of days (or possibly weeks), until the river should fall two or three feet, and let them get back to their log-cabin and their chills again—*chills being a merciful provision of an all-wise Providence, to enable them to take exercise without exertion.*

